# The presence of serum anti‐SARS‐CoV‐2 IgA appears to protect primary health care workers from COVID‐19

**DOI:** 10.1002/eji.202149655

**Published:** 2022-02-18

**Authors:** Viktoria Hennings, Karolina Thörn, Sofie Albinsson, Christine Lingblom, Kerstin Andersson, Christer Andersson, Katarina Järbur, Rille Pullerits, Manja Idorn, Søren R. Paludan, Kristina Eriksson, Christine Wennerås

**Affiliations:** ^1^ Department of Clinical Microbiology Region Västra Götaland Sahlgrenska University Hospital Göteborg Sweden; ^2^ Department of Rheumatology and Inflammation Research Sahlgrenska Academy Institute of Medicine University of Gothenburg Göteborg Sweden; ^3^ Department of Infectious Diseases Institute of Biomedicine Sahlgrenska Academy University of Gothenburg Göteborg Sweden; ^4^ Nötkärnan Primary Health Care Center Consortium Göteborg Sweden; ^5^ Department of Biomedicine Aarhus University Aarhus Denmark

**Keywords:** IgA, SARS‐CoV‐2, Cytotoxic T cell, Neutralizing antibodies, Primary health care

## Abstract

The patterns of humoral and cellular responses to SARS‐CoV‐2 were studied in Swedish primary health care workers (n = 156) for 6 months during the Covid‐19 pandemic. Serum IgA and IgG to SARS‐CoV‐2, T‐cell proliferation and cytokine secretion, demographic and clinical data, PCR‐verified infection, and self‐reported symptoms were monitored. The multivariate method OPLS‐DA was used to identify immune response patterns coupled to protection from Covid‐19. Contracting Covid‐19 was associated with SARS‐CoV‐2‐specific neutralizing serum IgG, T cell, IFN‐γ, and granzyme B responses to SARS‐CoV‐2, self‐reported typical Covid‐19 symptoms, male sex, higher BMI, and hypertension. Not contracting Covid‐19 was associated with female sex, IgA‐dominated, or no antibody responses to SARS‐CoV‐2, airborne allergy, and smoking. The IgG‐responders had SARS‐CoV‐2‐specific T‐cell responses including a cytotoxic CD4+ T‐cell population expressing CD25, CD38, CD69, CD194, CD279, CTLA‐4, and granzyme B. IgA‐responders with no IgG response to SARS‐CoV‐2 constituted 10% of the study population. The IgA responses were partially neutralizing and only seen in individuals who did not succumb to Covid‐19. To conclude, serum IgG‐dominated responses correlated with T‐cell responses to SARS‐CoV‐2 and PCR‐confirmed Covid‐19, whereas IgA‐dominated responses correlated with not contracting the infection.

## Graphical abstract

Immunity development to SARS‐CoV‐2 was monitored in primary care health workers for 6 months. IgA‐only responders appeared to be protected from contracting Covid‐19. Being an IgG responder was associated with contracting Covid‐19 and SARS‐CoV‐2‐specific T‐cell responsiveness. There was scant evidence of T‐cell responses to SARS‐CoV‐2 among seronegative individuals.

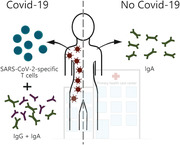

## Introduction

SARS‐CoV‐2, the virus that emerged in humans in 2019 and led to the Covid‐19 pandemic, is a respiratory virus that may cause acute respiratory distress syndrome and has a mortality rate of approximately 0.3% [1]. At the same time, SARS‐CoV‐2 gives rise to asymptomatic infections in at least 10% of infected individuals [2, 3]. The mechanisms underlying the spectrum of Covid‐19 symptoms remain unclear, but high age and male sex are important risk factors for morbidity and mortality [4, 5].

SARS‐CoV‐2 infection elicits robust B‐cell and T‐cell responses, especially in individuals with severe disease [6]. SARS‐CoV‐2‐specific antibodies appear within 1–2 weeks of infection in the vast majority of infected patients and memory B cells are present in Covid‐19 convalescents [7, 8, 9]. SARS‐CoV‐2‐specific IFN‐γ‐secreting CD4^+^ T cells and cytotoxic CD8^+^ T cells develop in both acute and asymptomatic SARS‐CoV‐2‐infections and persist as memory T cells after recovery [10, 11, 12, 13, 14].

The antibody response to SARS‐CoV‐2 reflects the fact that the virus enters and infects a mucosal surface. SARS‐CoV‐2‐specific IgA is often detected prior to SARS‐CoV‐2‐specific IgM and IgG [15, 16], and dominates the early neutralizing antibody response to SARS‐CoV‐2 in serum and saliva [15]. The SARS‐CoV‐2‐specific IgA responses are less diverse than the IgG responses and predominantly bind to the spike protein [15]. Sera of individuals who develop IgA, IgG, and IgM against SARS‐CoV‐2 have higher neutralizing capacity compared to sera from individuals who only develop IgG responses [8], and dimeric IgA, the primary form of IgA in the upper respiratory tract, is 15 times more potent than monomeric IgA in neutralizing SARS‐CoV‐2 [9].

The goal of this study was to investigate the patterns of humoral and cellular responses to SARS‐CoV‐2 in primary health care workers during the Covid‐19 pandemic. How serum IgA and IgG, as well as T‐cell responses to SARS‐CoV‐2 developed in relation to documented exposure to Covid‐19, demographic and clinical data, and self‐reported symptoms were analyzed by a multivariate method of pattern recognition. We found that serum IgG‐dominated responses correlated with T‐cell responses to SARS‐CoV‐2 and PCR‐confirmed Covid‐19, whereas IgA‐dominated responses correlated with not contracting the infection.

## Results

One hundred‐fifty of the 156 enrolled study participants completed the entire study. The mean age of the study participants was 44 (range 22–70) and 79% were women. The most common medical conditions were airborne allergy (22%), migraine (5.8%), autoimmune disease (5.1%), hypertension (4.5%), diabetes (1.9%), and 2.6% of the study participants were immunocompromised (Supporting information Table S1). The participants joined the study in April or May of 2020. The study period coincided with the first and second waves of Covid‐19 in the Västra Götaland region of Sweden, which began in March and October of 2020, respectively. Sixteen study participants (10%) contracted Covid‐19 verified by PCR during the study period, and six study participants (3.8%) cohabited with a SARS‐CoV‐2 PCR‐positive person. However, it must be emphasized that PCR‐testing was not available in Västra Götaland until May or June 2020 for persons with suspected Covid‐19 who were not hospitalized. None of the study participants required hospitalization for Covid‐19.

### Antibody responses to SARS‐CoV‐2

**Figure 1:**
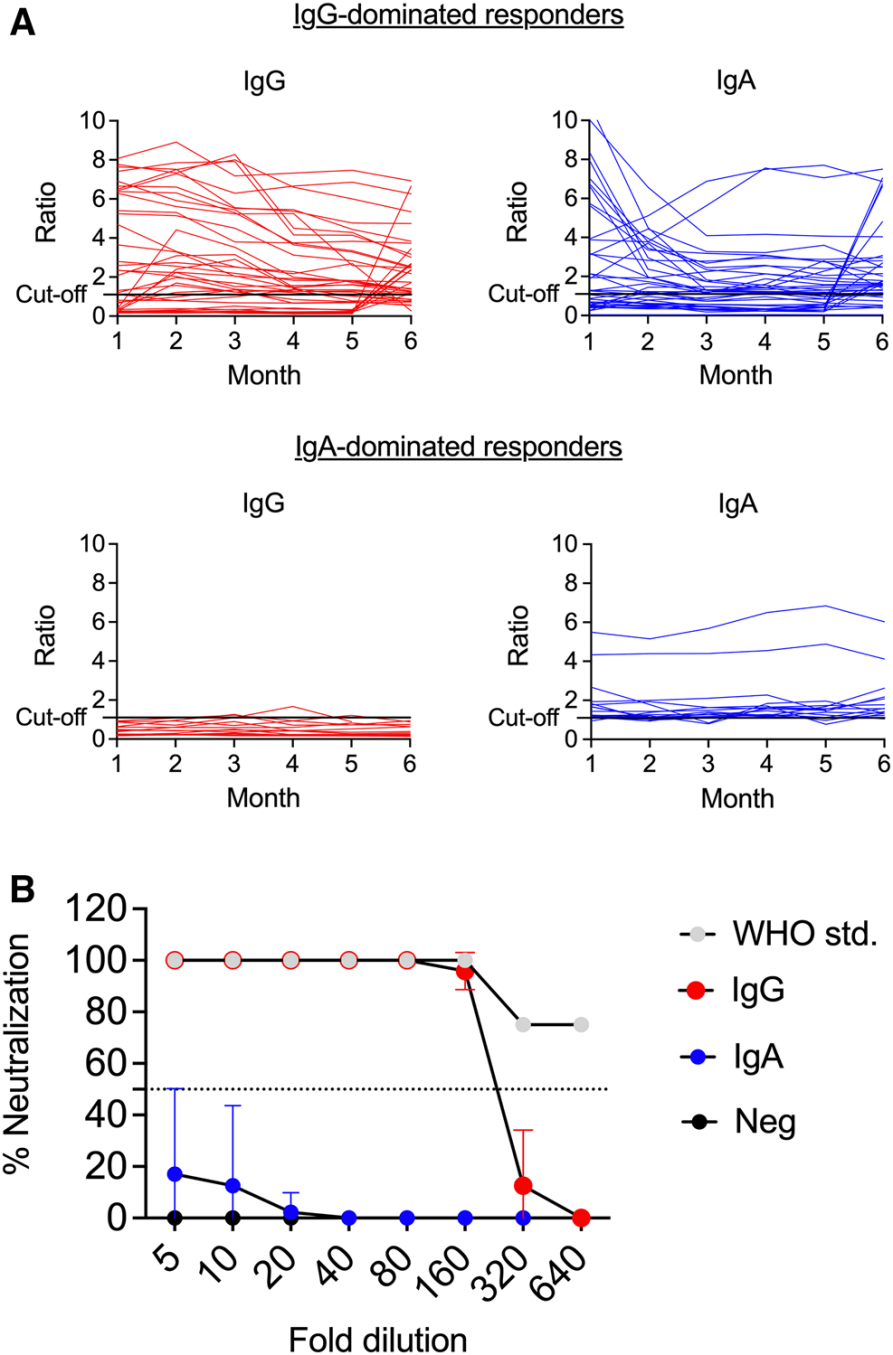
**IgG‐dominated versus IgA‐dominated humoral responses and SARS‐CoV‐2 neutralizing capacity**. (A) Serum samples were collected once a month during a period of 6 months. Levels of IgG and IgA antibodies to SARS‐CoV‐2 were analyzed using ELISA. Data are presented as ratios, which corresponds to an adjusted OD‐value (OD‐value of serum sample/OD‐value of calibrator), over time in IgG‐dominated and IgA‐dominated responders. A ratio of ≥1.1 was positive; ≥0.8 to <1.1 borderline; <0.8 negative. (B) Neutralization capacity of sera from patients positive for anti‐SARS‐CoV‐2 IgG (red, n = 3), IgA‐dominated responders (blue, n = 11) and from patients with no antibodies against SARS‐CoV‐2 (black, n = 3) was tested using a live virus PRNT neutralization assay. The WHO anti‐SARS‐CoV‐2 IgG standard (grey) was included as a positive control. Percent antibody‐mediated neutralization of the Freiburg SARS‐CoV‐2 isolate was measured at 10‐point twofold serum dilutions (8 points depicted). Data are presented as mean with standard deviation.

All study participants were monitored monthly for 6 months for serum IgA and IgG antibodies to the spike protein of SARS‐CoV‐2. One third of the study participants (53/150) had detectable IgA and/or IgG antibodies to the SARS‐CoV‐2 spike protein, half of the participants were negative throughout the study, and the remainder had borderline levels of antibodies. We divided the participants into three groups depending on antibody pattern: (1) antibody responses dominated by IgG, that is, IgG either alone or in combination with IgA (n = 38), (2) antibody responses dominated by IgA, that is, IgA either alone or in combination with borderline IgG responses or occasional IgG responses (n = 15), and (3) negative antibody response, which also included borderline IgA responses (n = 97) (Fig. 1A). Serum from IgA responders and from a few IgG responders who had tested positive for SARS‐CoV‐2 by PCR were analyzed using an in vitro SARS‐CoV‐2 neutralization assay. IgA‐only sera (n = 11) partially neutralized SARS‐CoV‐2 whereas the IgG‐positive sera (n = 3) fully neutralized SARS‐CoV‐2 with 50% neutralization obtained at serum dilutions ranging between 5 and 320 (Fig. 1B).

### T‐cell responses to SARS‐CoV‐2

**Figure 2:**
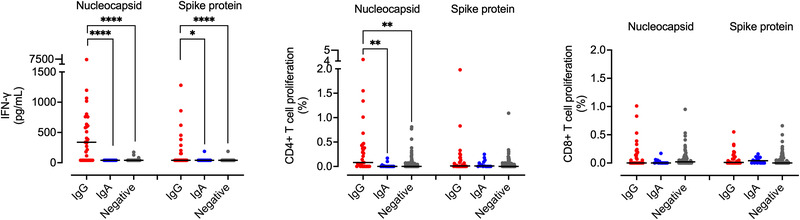
**T‐cell proliferation and IFN‐γ responses evoked by SARS‐CoV‐2 peptides**. PBMC from IgG‐dominated responders (n = 33), IgA‐dominated responders (n = 15), and antibody‐negative individuals (n = 93) were incubated for 5 days with nucleocapsid peptides, spike protein peptides, or medium alone. IFN‐γ levels in cell supernatants were measured by ELISA. Proliferation of CD4+ and CD8+ T cells in response to peptides was determined by flow cytometry analysis of bleaching of viability‐stained T cells and is indicated as % of the total population of CD4+ and CD8+ T cells that proliferated upon stimulation with viral peptides minus the fraction of spontaneously proliferating T cells (in medium alone). Data are presented as scatter dot plots with horizontal median lines. Kruskal–Wallis nonparametric test with Dunn's post‐test. *p < 0.05, **p < 0.01, ***p < 0.001, and ****p < 0.0001. Absence of asterisks indicates nonsignificant results.

**Figure 3:**
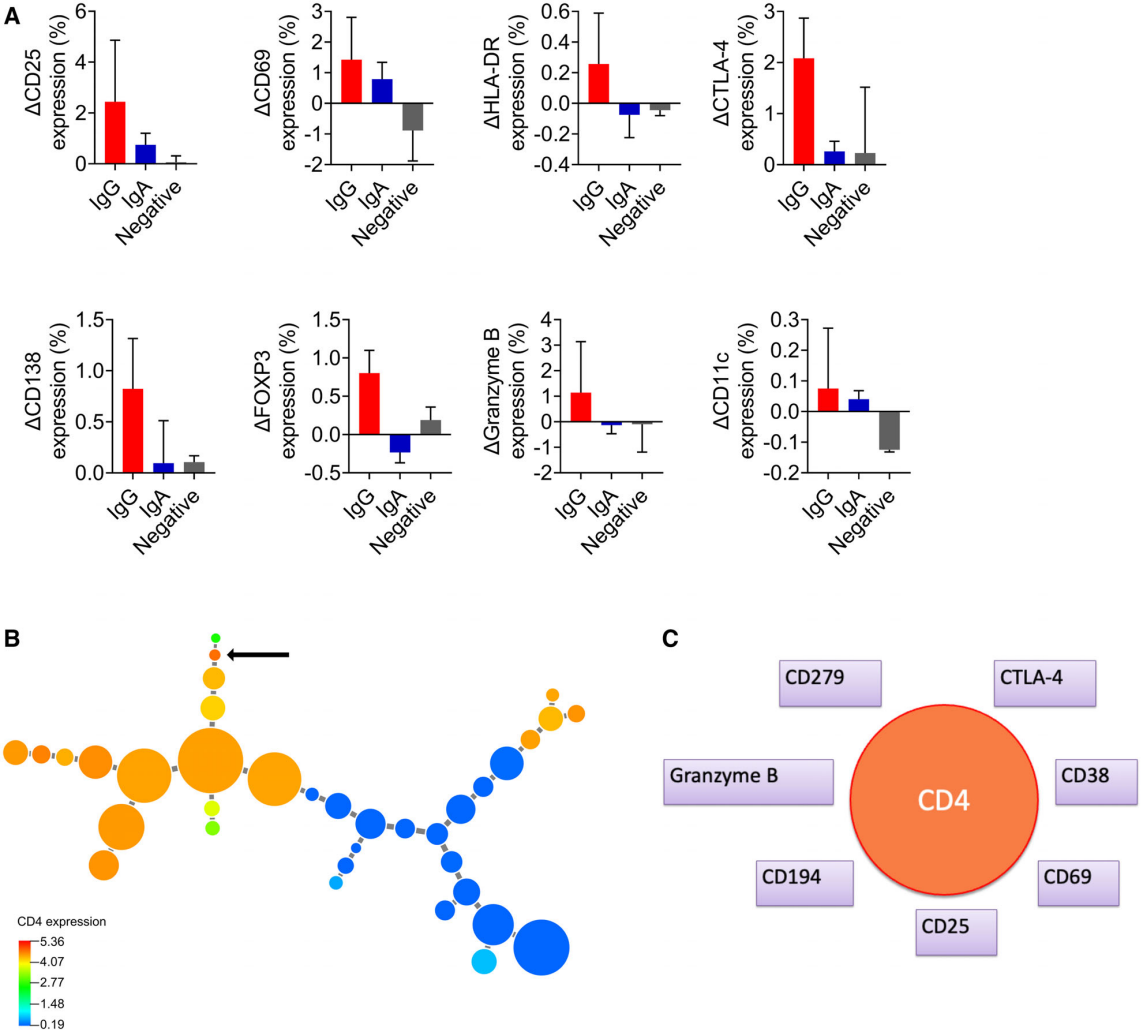
**T‐cell populations that responded to stimulation with SARS‐CoV‐2 peptides**. PBMC from individuals with IgG‐dominated, IgA‐dominated, and no antibody response to Covid‐19 were stimulated with a mixture of spike protein and nucleocapsid peptides and analyzed for the expression of 35 extracellular and intracellular molecules by CyTOF. (A) Graphs indicating the eight molecules associated with an IgG‐dominated response based on results obtained from an Orthogonal‐Projection to Latent Structures Discriminatory Analysis (OPLS‐DA, data not shown). IgG‐dominated responders (red, n = 4), IgA‐only responders (blue, n = 2), and antibody nonresponders (grey, n = 2). Data are presented as mean with standard deviation. (B) Minimum spanning tree composed of CD3+ T‐cell populations derived from IgG‐dominated responders (n = 4) and IgA‐only responders (n = 2) that were stimulated or sham‐stimulated with the mixture of spike protein and nucleocapsid peptides. The arrow indicates a CD4+ T‐cell population that was only seen among the IgG‐dominated responders after 5 days of stimulation with viral peptides. The size of the circles indicates the relative sizes of the cell populations and their coloring shows the relative intensity of CD4 expression. (C) Phenotype of the T‐cell population indicated by an arrow in *B*.

IFN‐γ production, along with proliferation of CD4^+^ T cells in response to stimulation with nucleocapsid‐derived peptides of SARS‐CoV‐2 was characteristic of individuals with IgG antibody responses to Covid‐19 (Fig. 2). The IgG responders also had T cells that produced IFN‐γ to spike protein‐derived peptides (Fig. 2). In contrast, the IgA responders had virtually no IFN‐γ response, nor CD4 T‐cell proliferative response to the virus (Fig. 2). More detailed multiplex analyses of cytokines and other immune mediators revealed that PBMC from the IgG responders produced significantly elevated levels of IL‐2, granzyme B, IL‐10, CD40L, IFN‐γ, MIP‐1‐α, TNF‐α, MCP‐1, IP‐10, and GM‐CSF in response to nucleocapsid‐derived peptides compared to leukocytes from antibody‐negative individuals and IgA‐only responders (Supporting information Fig. S1). Overall, the IgA‐dominated responders had limited T‐cell responses to SARS‐CoV‐2. Individuals with the IgG‐responder profile (n = 4), IgA‐only profile (n = 2), and without antibodies to SARS‐CoV‐2 (n = 2) were selected for analysis of T‐cell expression of 28 molecules by Cytometry by time‐of‐flight (CyTOF) (Supporting information Tables S2, S3). SARS‐CoV‐2 peptide‐stimulated T cells from the IgG group upregulated their expression of the activation markers CD25, CD69, and HLA‐DR, the costimulatory molecules CTLA‐4 and CD138, the transcription factor FOXP3, cytotoxic granzyme B and integrin CD11c, which was not seen for T cells from the IgA‐dominated or antibody‐negative groups (Fig. 3A). Clustering analysis by employing the X‐shift algorithm in VorteX revealed a unique revealed a unique T‐cell population specific for SARS‐CoV‐2 that was exclusive for the IgG group: out of 60,000 analyzed T cells, 65 cells (0.11% of the T‐ cell population) belonged to this cluster and all but one cell were derived from the IgG‐responder group (Fig. 3B). This T‐cell population expressed CD4, CTLA‐4, CD38, CD69, CD25, CD194, granzyme B, and CD279 (Fig. 3C).

### Clinical and immunological correlates of antibody patterns

**Figure 4:**
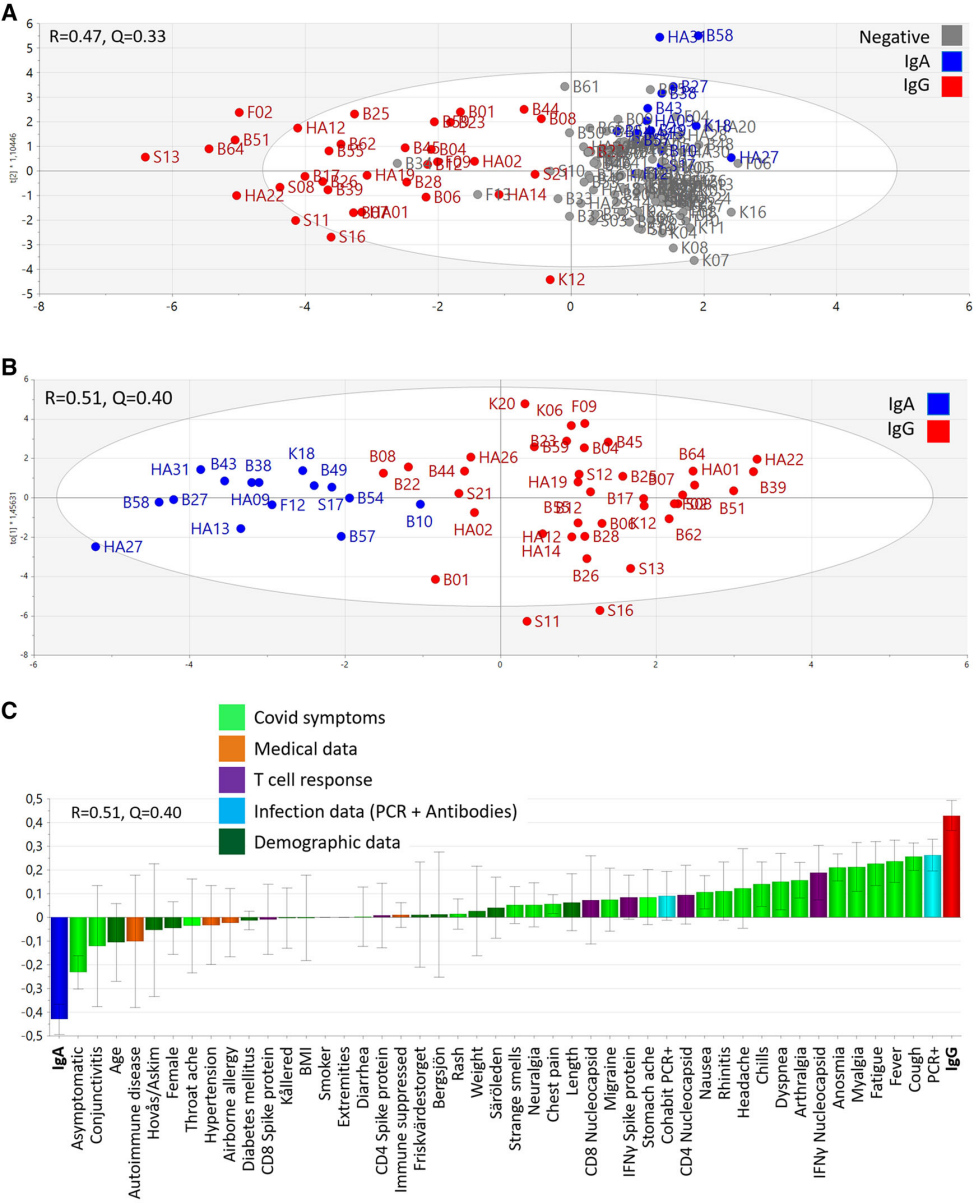
**Clustering of individuals with IgG‐dominated and IgA‐dominated types of antibody responses**. The multivariate method “Orthogonal‐Projection to Latent Structures Discriminatory Analysis” (OPLS‐DA) was used to examine if the study parameters (X‐variables) could separate individuals with the various antibody patterns (Y‐variables). (A) A multivariate model showing the clustering of individuals with IgG‐dominated antibody responses (red, n = 38), IgA‐dominated antibody responses (blue, n = 15), and no antibody response (grey, n = 97). Each symbol denotes one individual. The model's stability (Q) and explanatory power (R) is indicated. (B) A multivariate model shows segregation of individuals with the IgG‐dominated antibody pattern of response (red, n = 38) from individuals with IgA‐dominated antibody response (blue, n = 15). The model's stability (Q) and explanatory power (R) is indicated. (C) A loading plot of the OPLS‐DA model depicted in *B* shows which of the study parameters that had the largest impact on the separation of the IgG‐dominated responders from the IgA‐only responders. The study parameters (X‐variables) were grouped into the categories demographic data (dark green), medical data (orange), infection data (light blue), Covid‐19 symptoms (light green) and T‐cell responses (purple). Variable bars that are close to and point in the same direction as the bars indicating type of antibody pattern are positively associated with said antibody pattern.

We next examined if the three antibody patterns (IgG‐dominated, IgA‐dominated, and negative) were associated with PCR‐verified Covid‐19, Covid‐19 symptoms, T‐cell responses to SARS‐CoV‐2, demographic parameters (age, sex, weight, BMI, primary healthcare center), and comorbidities by making a multivariate Orthogonal Projections to Latent Structures by means of Partial Least Squares Discriminant Analysis (OPLS‐DA) model. This approach rendered a reasonably good model with an explanatory power of 47% (R2Y = 0.47) and acceptable stability (Q2Y = 0.33). The cluster corresponding to the IgG‐dominated response was more separate from the IgA‐dominated response than from the negative cluster (Fig. 4A). Therefore, we made a second model composed of these two antibody patterns, the IgG‐dominated response, and the IgA‐dominated response. This model turned out to be stable (Q2Y = 0.40) with an explanatory power of 51% (R2Y = 0.51), which indicates that these two responder types differed significantly from one another (Fig. 4B). Figure 4C shows that the IgA‐only responders were more often asymptomatic or had conjunctivitis and/or throat ache compared with the IgG group. The IgA group also tended to be older and female with underlying autoimmune conditions, airborne allergy, and hypertension. The association of IgA‐only responses with autoimmunity and a study documenting false‐positive SARS‐CoV‐2 antibody tests in autoimmune individuals [17] prompted us to exclude that this was artefact caused by rheumatoid factor (RF). However, none of the IgA‐dominated responders were positive for IgA or IgM RF (data not shown). Conversely, being an IgG‐responder was associated with PCR‐positive Covid‐19 and cohabitation with a PCR‐positive person and, in descending order of importance, symptoms cough, fever, fatigue, myalgia, anosmia, arthralgia, dyspnea, chills, headache, rhinitis, and nausea (Fig. 4C). IFN‐γ production and CD4^+^ T‐cell proliferation to nucleocapsid‐derived peptides was also a feature of being an IgG‐responder (Fig. 4C).

### Clinical and immunologic correlates of verified SARS‐CoV‐2 infection

**Figure 5:**
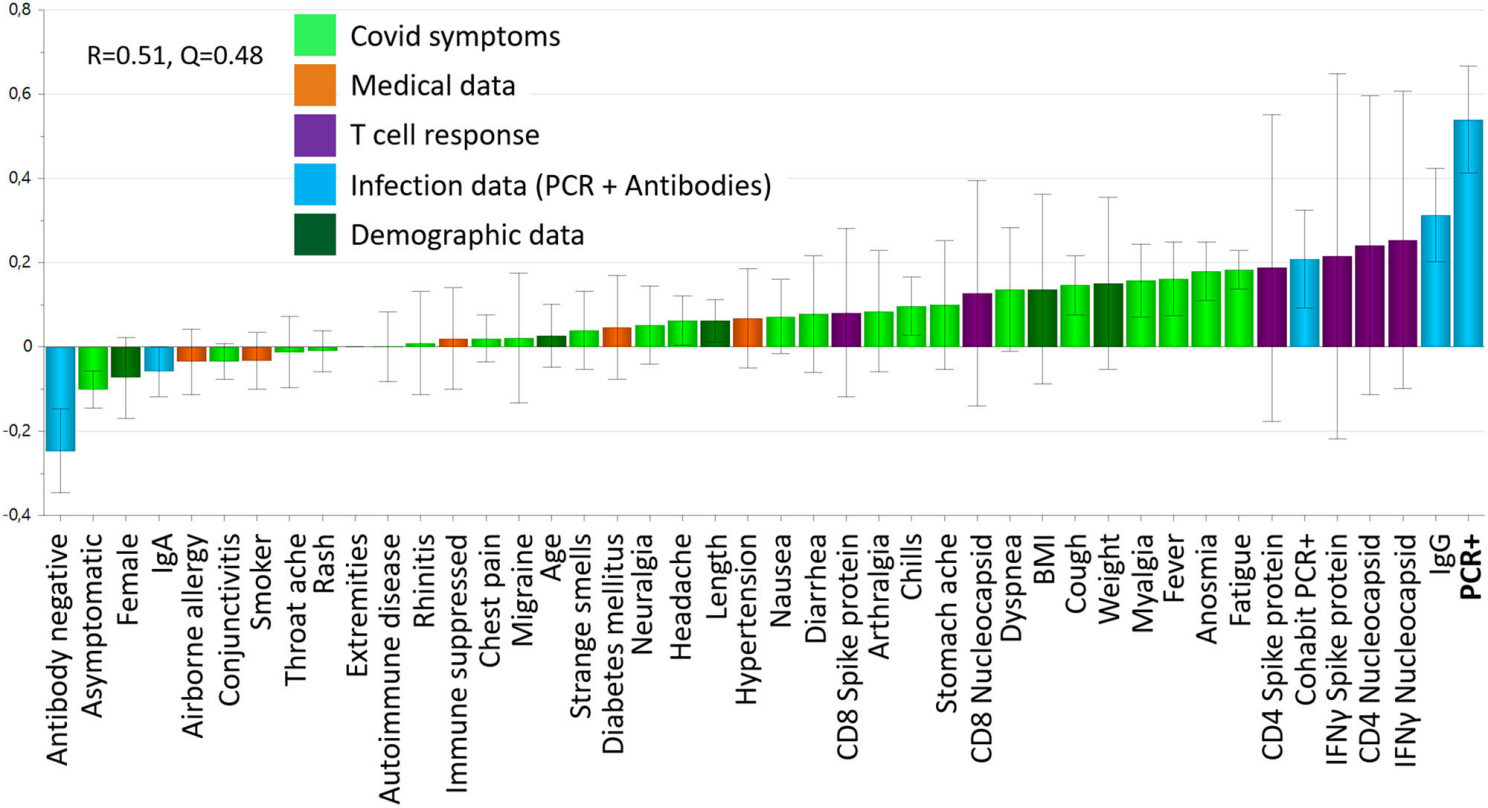
**Correlates of PCR‐positivity to SARS‐CoV‐2**. Multivariate analyses were made using the Orthogonal‐Projection to Latent Structures method (OPLS) followed by Variable Importance in the Projection (VIP) analysis with a cut‐off of 0.5. Loading plot (n = 150) depicting the relationship between having tested positive for SARS‐CoV‐2 by PCR with the study parameters Covid‐19 symptoms (light green), medical data (orange), T‐cell response (purple), infection data (light blue) and demographic data (dark green). The quality of the model is indicated by its stability (Q) and explanatory power (R). Variable bars that are close to and point in the same direction as the “PCR+” bar are positively associated and bars that point in the opposite direction are negatively associated with PCR‐positivity.

Last, we made a multivariate model to establish which of the studied clinical, demographic, and immune parameters were associated with confirmed SARS‐CoV‐2 infection. Individuals who had tested positive for SARS‐CoV‐2 by PCR more frequently cohabited with persons who also had tested positive by PCR, had the IgG type of antibody response, featured IFN‐γ production and CD4+ T‐cell proliferation to nucleocapsid and to spike proteins, more often self‐reported fatigue, anosmia, fever, myalgia, cough, and dyspnea and tended to have higher body weight and BMI (Fig. 5). PCR‐positivity was inversely associated with being female, asymptomatic, and either having no antibodies to SARS‐CoV‐2 or having the IgA‐response pattern. Airborne allergy and smoking were also more frequent among individuals who did not test positive for SARS‐CoV‐2 by PCR. This model had an explanatory power of 51% (R2Y = 0.51) and good stability (Q2Y = 0.48) (Fig. 5).

## Discussion

The main goal of this prospective study was to couple the antibody and T‐cell responses to SARS‐CoV‐2 with demographic parameters and clinical features of Covid‐19. We chose to study a relatively healthy group of people, primary health care workers naturally exposed to SARS‐CoV‐2, for a period of 6 months during the Covid‐19 pandemic. Our study cohort was representative of health care workers in Sweden, with the exact same mean age of 44, similar female predominance (our study 79% versus 85%) and IgG seroprevalence to SARS‐CoV‐2 (23% versus 19%) as a larger cross‐sectional study conducted among hospital employees in Sweden in the spring 2020 [18]. We identified two main patterns of immune responses to SARS‐CoV‐2: an IgG‐dominated and an IgA‐dominated pattern. Only individuals with IgG responses developed T‐cell responses to SARS‐CoV‐2. IgG responsiveness was associated with SARS‐CoV‐2 PCR positivity and self‐reported typical Covid‐19 symptoms. In contrast, IgA responsiveness was associated with limited T‐cell responses to SARS‐CoV‐2, autoimmunity, airborne allergy, and not contracting Covid‐19.

SARS‐CoV‐2 IgA‐only responders constituted 10% of our cohort which is in line with other studies [8, 19], and 87% of them were already IgA‐positive at the start of the study. It is possible that this IgA response constituted cross‐reactive IgA antibodies generated in response to other coronaviruses, even though the S1 subunit of the SARS‐CoV‐2 spike protein used in our antibody tests is less conserved among different Coronavirus strains compared with the S2 subunit [20]. Interestingly, none of the IgA‐only responders reported any Covid‐19‐associated symptoms nor had PCR‐confirmed SARS‐CoV‐2 infection, which implies that SARS‐CoV‐2‐specific IgA‐responses may protect against contracting Covid‐19. Indeed, one‐third of the SARS‐CoV‐2‐specific serum IgA‐dominated sera partially neutralized the virus in vitro. It is known that serum IgA is less abundant than serum IgG and not as efficient as serum IgG and mucosal IgA at neutralizing SARS‐CoV‐2 [9]. The IgA‐producing plasma cells that produce serum IgA and mucosal IgA usually originate from the same B‐cell clones, but serum IgA is mainly monomeric and consequently of lower avidity compared to mucosal IgA, which is mostly dimeric and predominantly of the IgA2 subclass [21]. The serum IgA we have monitored in this study may be said to be a surrogate marker of nasal IgA, the latter of which confers protection from Covid‐19 by preventing virus entry into the body. A limitation of our study is that we did not investigate corresponding nasal IgA antibody levels to SARS‐CoV‐2 and their neutralizing capacity.

Contrary to the study of Sekine et al., we did not find clear‐cut antiviral T‐cell responses in person without antibodies to SARS‐CoV‐2 although we used the same SARS‐CoV‐2‐spike protein peptides to stimulate the T cells in vitro [14]. A likely explanation is that we abstained from adding the T‐cell growth factor IL‐2 and crosslinking the costimulatory molecules CD28/CD49d in our experimental setup.

Our most interesting finding relating to SARS‐CoV‐2 T‐cell responses was the detection of a unique virus‐specific cytotoxic CD4^+^ T‐cell population only harbored by individuals who responded with serum IgG to SARS‐CoV‐2. The virus‐specific T‐cell population expressed the activation markers CD25, CD38, and CD69, the inhibitory molecules CTLA‐4 and CD279 (PD‐1), cytotoxic granzyme B, and the chemokine receptor CCR4 (CD194). Upregulation of inhibitory molecules, such as CTLA‐4 and PD‐1, by a SARS‐CoV‐2‐specific T‐cell subset reflects highly activated effector T cells capable of producing large quantities of granzyme B and IFN‐γ [22].

Several of the demographic and clinical parameters revealed in the multivariate analyses to be associated with contracting PCR‐verified Covid‐19 are well‐established risk factors for severe Covid‐19 such as male sex and higher BMI. However, none of our study participants required hospitalization for Covid‐19. Nevertheless, male sex is a risk factor for most infectious diseases [23]. We identified female sex, airborne allergy, and smoking to be associated with protection from Covid‐19, which is in line with previous findings. A Spanish registry study covering close to half a million individuals showed that the risk of contracting Covid‐19 was lower in asthmatics with an odds ratio of 0.74 (95% CI: 0.71–0.77) [24]. Although it is clear that smoking is a risk factor for the severity of Covid‐19, early studies reported an underrepresentation of smokers among patients hospitalized for Covid‐19 [25]. Perhaps smoking‐induced inflammation of the upper respiratory mucosa provides low‐degree protection against transmission of viral infection.

Our study attempted to cover a gap in knowledge regarding how immunity to SARS‐CoV‐2 develops over time in a relatively healthy group of adults, and how this relates to the risk of becoming infected, demographic, and clinical risk factors, and immune correlates of protection from contracting Covid‐19. The vast majority of published studies on Covid‐19 have been cross‐sectional and/or focused on hospitalized patients with severe disease. Our key findings were that (1) every tenth person had a potentially neutralizing IgA response which was associated with not contracting Covid‐19; (2) an IgG response was strongly associated with T‐cell responsiveness to SARS‐CoV‐2 and having contracted Covid‐19, and (3) there was scant evidence of T‐cell responsiveness to SARS‐CoV‐2 among seronegative individuals.

## Materials and methods

### Study design

Health care workers employed at one of five primary health care centers in the Gothenburg area of Sweden (Nötkärnan Primary Care Health Centers of Bergsjön, Kållered, Friskväderstorget, Hovås Askim, and Säröleden) were recruited to the study. Study participants donated 5 mL of blood every month for 6 months starting in April or May 2020. Self‐reported demographic data (age, sex, length, body weight, profession, postal address, number of persons in household, cohabitation with household member with PCR‐verified or suspected Covid‐19, close work with colleague who had PCR‐verified or suspected Covid‐19) and medical history (medical conditions, medication, immune suppression, smoking habits) were collected from all participants at the start of the study. Study participants who had antibodies to SARS‐CoV‐2 at the start of the study answered written questions regarding the suspected mode of transmission (via household member, colleague at work, patient, or other) and if they had tested positive for Covid‐19 by PCR and were asked to fill in a questionnaire about Covid‐19 symptoms (Supporting information Fig. S2). All participants who developed suspected Covid‐19 symptoms during the study period were asked to fill in the same questionnaire and to take a Covid‐19 PCR test. Participants with serum antibodies to SARS‐CoV‐2 at the start of the study or who seroconverted during the study were asked to donate heparin‐anticoagulated blood (20 mL) 1–2 months later, which was processed and frozen for later T‐cell analyses. All study participants, including those who never seroconverted to SARS‐CoV‐2, were asked to donate blood for T‐cell analyses at the end of the study. Written consent was obtained from all who participated in the study, which was approved by the Swedish Ethical Review Authority (Dnr 2020–02962).

### Antibodies

Serum IgA and IgG antibodies to the S1 domain of the spike protein of SARS‐CoV‐2 were determined using Euroimmun Anti‐SARS‐CoV‐2 ELISA kit IgG and IgA (Lübeck, Germany). Antibody levels are reported as ratios (OD‐value of serum sample/OD‐value of calibrator). A ratio of ≥1.1 was positive; ≥0.8 to <1.1 borderline; <0.8 negative.

### T‐cell responses

Within 12 h of blood sampling, PBMCs were isolated by density gradient centrifugation and stored frozen at −140°C until use. Thawed cells were stained with Celltrace Violet (Thermo Fisher, Waltham, MA, USA) and diluted in X‐Vivo 15 culture medium with gentamicin. Cells (2 × 10^5^) were seeded into 96‐well TC‐plates (Sarstedt, Nümbrecht, Germany) and incubated in triplicate with viral peptides from the SARS‐CoV‐2 spike protein (S peptides) and nucleocapsid (N peptides), respectively (both Miltenyi Biotec, Bergisch Gladbach, Germany) at a final concentration of 0.2 μg/mL. PHA (5 μg/mL, Roche, Basel, Switzerland) was used as positive control, and culture medium as negative control. On day 5, the cells and supernatants were collected. Supernatants were frozen at −80°C for later analyses. The cells were stained with mouse IgG1, κ anti‐human antibodies; CD4 APC (clone SK3), CD8 PE‐Cy7 (clone RPA‐T8), and CD3 FITC (clone UCHT‐1) (all from BD Bioscience Franklin Lakes, NJ, USA) diluted in FACS buffer (PBS with 2 mM EDTA + 2% FBS; 41 μL). The cells were washed, resuspended in 200 μL FACS buffer, and stained with 10 μL of 7AAD (BD Bioscience) 10 min before flow cytometry analysis using a FACSLyric instrument (BD Bioscience) equipped with FACSuite software (BD Bioscience). Flow cytometry data were analyzed using FlowJo software (version 10.7.1, Tree Star, Ashland, OR, USA). The gating strategy used to identify proliferating CD4^+^ and CD8^+^ T cells is shown in Supporting information Fig. S3. Proliferation to viral peptides was expressed as percent proliferating T cells after subtraction of spontaneous proliferation in negative control wells. We have adhered to the guidelines for the use of flow cytometry and cell sorting in immunological studies described by Cossarizza et al [26].

### Immune markers

The ELISA kit Human IFN‐γ Duo set (R&D Systems, Minneapolis, MI, USA) was used to determine levels of IFN‐γ in cell supernatants. Twenty‐four cytokines and other immune markers (CD40 ligand, GM‐CSF, granzyme B, IFN‐α, IFN‐γ, IL‐1α, IL‐1β, IL‐1RA, IL‐2, IL‐4, IL‐6, IL‐8, IL‐10, IL‐12p70, IL‐13, IL‐15, IL‐17A, IL‐33, IP‐10, MCP‐1, MIP‐1α, MIP‐1β, PD‐L1, TNF‐α) were quantified in supernatants from PBMC exposed for 5 days to N peptides using Human Discovery Immunotherapy Magnetic Luminex Performance Assay 24‐plex Fixed Panel (R&D Systems).

### CyTOF

PBMC (n = 8) were thawed, seeded into 96‐well TC‐plates (2 × 10^5^ cells/well), and incubated with a mixture of N and S peptides (0.2 μg/mL/peptide) in X‐Vivo 15 medium or medium alone (5 days, 37°C, 5% CO_2_). The cells were washed, incubated with 5 μM Cell ID Cisplatin (Fluidigm, South San Francisco, CA, USA), washed, and incubated (30 min, RT) with an antibody cocktail against extracellular markers (Supporting information Table S3) and 7% Human TruStain FcX (BioLegend, San Diego, CA, USA). The samples were washed, fixed with 1.6% formaldehyde, and permeabilized using Foxp3/Transcription Factor Staining buffer (eBioscience, San Diego, CA, USA) before incubation with antibodies directed against intracellular markers (Supporting information Table S3). The cells were washed, incubated with 62.5 nM Cell‐ID Intercalator‐Ir diluted in Maxpar Fix and Perm Buffer (Fluidigm, 45 min, RT), and stored at −80°C until analysis using a Helios mass cytometer with CyTOF Software version 7.0. (Fluidigm) and gated using Flow Jo. Clustering analysis was done on gated CD3^+^ T cells (Supporting information Fig. S4) using the X‐shift algorithm of VorteX software version 29/06/17 [27].

### Neutralization assay

The SARS‐CoV‐2 Freiburg isolate FR‐4286, kindly provided by Professor Georg Kochs, University of Freiburg, was propagated in VeroE6 cells expressing human TMPRSS2 (VeroE6‐hTMPRSS2) as described [28]. Serum samples were heat‐inactivated (30 min, 56°C) and serially diluted in DMEM (Gibco), 2% FCS (Sigma‐Aldrich), 1% Penicillin/Streptomycin (Gibco), 1% l‐Glutamine (Sigma‐Aldrich). The first WHO International Standard Anti‐SARS‐CoV‐2‐IgG (NIBSC 20/136) was included as positive control. Sera were mixed with SARS‐CoV‐2 at a final titer of 100 TCID_50_/well (tissue culture infective dose 50%) and incubated at 4°C overnight. Next, virus:serum mixtures were added to 2 × 10^4^ VeroE6 TMPRSS2 cells seeded in flat‐bottom 96‐well plates, and incubated for 72 h at 37°C in 5% CO_2_, before 5% formalin fixation (Sigma‐Aldrich) and staining with crystal violet solution (Sigma‐Aldrich). The plates were read using a light microscope (Leica DMi1) with camera (Leica MC170HD) at 4× magnification, and the cytopathic effect was scored.

### Detection of Rheumatoid Factor

RF IgA and IgM isotype analyses were done using the EliA immunoassay (Phadia GmbH, Uppsala, Sweden).

### Statistics

Multivariate analyses of pattern recognition Orthogonal Projections to Latent Structures by means of Partial Least Squares Discriminant Analysis (OPLS‐DA) and OPLS were performed using the SIMCA‐P (version 15.0.2) statistical package (MKS Data Analytics Solutions, Malmö, Sweden). The models are given a value for explanatory power or goodness of fit, R and a value for stability, Q [29]. The Variable Importance Parameter was used as previously described [30]. Wilcoxon matched‐pairs signed rank test was used to compare two groups, and one‐way ANOVA Kruskal‐Wallis nonparametric test with Dunn's post‐test to compare three groups, applying GraphPad Prism software 9.0.2 (GraphPad, San Diego, CA, USA). A *p*‐value <0.05 was statistically significant.

## Author Contributions

CW and KE designed the study and obtained financial support. VH and KT handled laboratory logistics. VH, KT, KA, SA, MI, SP, and RP did the experimental work. CW, KE, VH, KT, SA, KA, MI, SP, and CL analyzed the data. CW, KE, VH, SA, and CL did the statistical analyses. CA and KJ were responsible for the study participants. KE, CW, and VH wrote the manuscript with revisions from all authors.

## Grants

The Swedish state under the agreement between the Swedish government and the county councils, the ALF‐agreement (ALFGBG‐722141, ALFGBG‐827291, ALFGBG grant for heavy equipment SU 2018‐01348), the Swedish Research Council (2020‐01287, 2020–02732), Cancer and Allergy Foundation (2020‐10154), Inga Britt and Arne Lundberg Research Foundation, the University of Gothenburg, the Swedish Cancer Foundation (CAN 2018/465), Independent Research Fund Denmark (0214‐00001B).

## Conflict of Interest

The authors have declared that no financial or commercial conflict of interest exist.

### Peer review

The peer review history for this article is available at https://publons.com/publon/10.1002/eji.202149655.

**Abbreviations**
OPLS‐DA: Orthogonal Projections to Latent Structures by means of Partial Least Squares Discriminant Analysis
RF: rheumatoid factor

## Supporting information

Supporting information

## Data Availability

The data that support the findings of this study are available from the corresponding author upon reasonable request.
