# Supplementary material for: The presence of serum anti‐SARS‐CoV‐2 IgA appears to protect primary health care workers from COVID‐19
Source: Eur J Immunol. 2022 Feb 18;52(5):800–9. doi: 10.1002/eji.202149655 (PMC9087394; doi:10.1002/eji.202149655)
Supplement: Supplementary file 1 — Supporting information [file EJI-52--s001.pdf]

Supplemental Figure 1

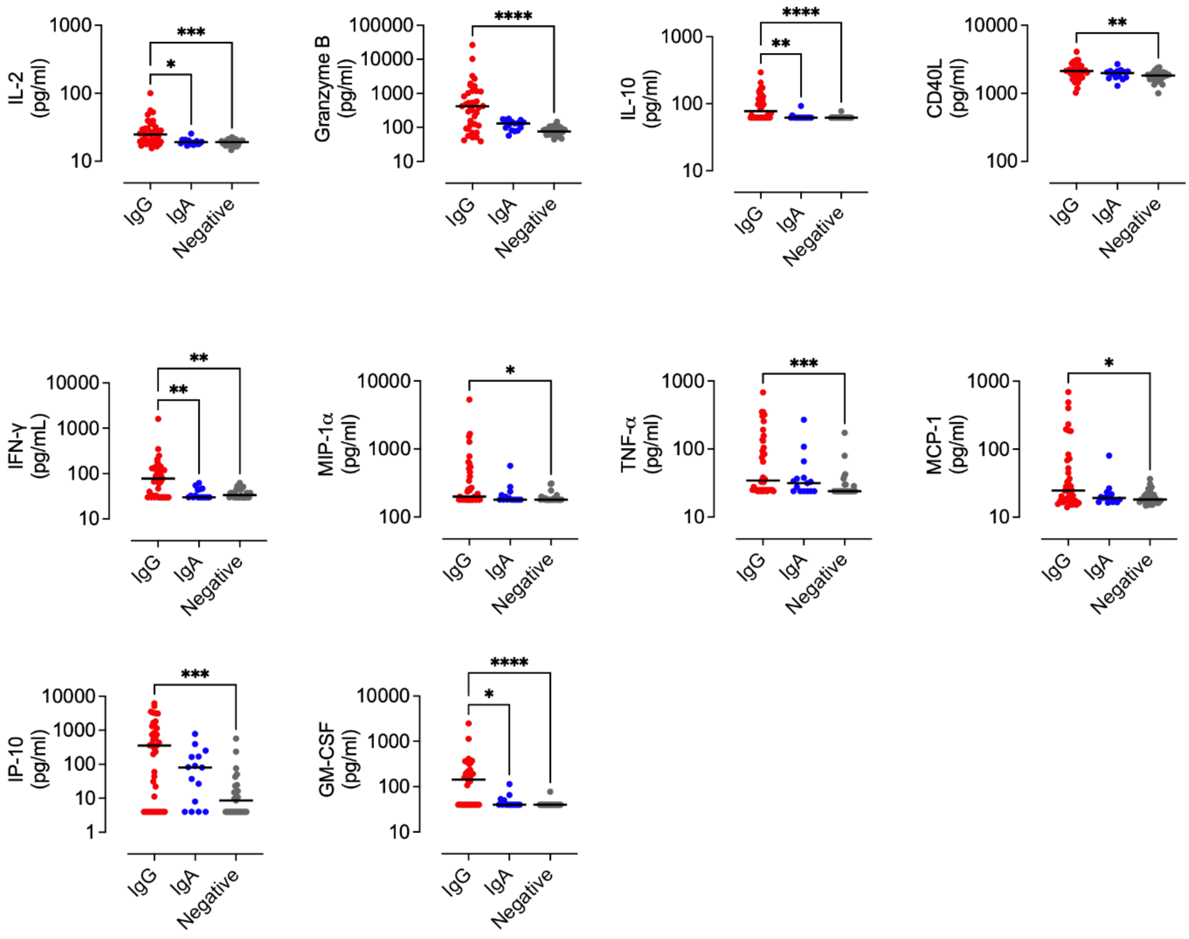

Supplemental Figure 1: Cytokines and immune mediators produced by T cells stimulated with nucleocapsid-derived peptides in individuals with IgG-dominated, IgA-dominated and no humoral responses. Levels of IL-2, granzyme B, IL-10, CD40L, IFN-γ, MIP-1α, TNF-α, MCP-1, IP-10 and GM-CSF were analyzed in PBMC supernatants from IgG-dominated (red, n=38), IgA-dominated (blue, n=15) and seronegative (grey, n=27) study participants after incubation with nucleocapsid peptides for 5 days. Data are presented as scatter dot plots with median lines. Kruskal-Wallis' non-parametric test with Dunn's post-test. *$p<0.05$, **$p<0.01$, ***$p<0.001$ and ****$p<0.0001$. Absence of asterisks indicates non-significant results.

Supplemental Figure 2

Covid-19 Symptoms Questionnaire. Translated from Swedish.
Check the boxes that fit your symptoms.

- ☐ Fever (> 38°C)
- ☐ Chills
- ☐ Feeling cold/frozen
- ☐ Tiredness (Fatigue)
- ☐ Headaches
- ☐ Muscular pain (Myalgia)
- ☐ Joint pain (Arthralgia)

- ☐ Runny or plugged nose (Rhinitis)
- ☐ Cough If yes, dry or purulent?:________________________
- ☐ Throat ache
- ☐ Red eyes (Conjunctivitis)
- ☐ Breathlessness (Dyspnea)
- ☐ Chest pain/pressure

- ☐ Skin rash If yes, which part of the body:______________________
- ☐ Discolored fingers or toes (Extremities)

- ☐ Stomach ache
- ☐ Nausea
- ☐ Diarrhea

- ☐ Unable to smell or taste (Anosmia)
- ☐ Strange smells or taste sensation (Smell)
- ☐ Numbness or pins and needles in hands or feet (Neuralgia)

- ☐ Other symptoms: ____

Supplemental Figure 3

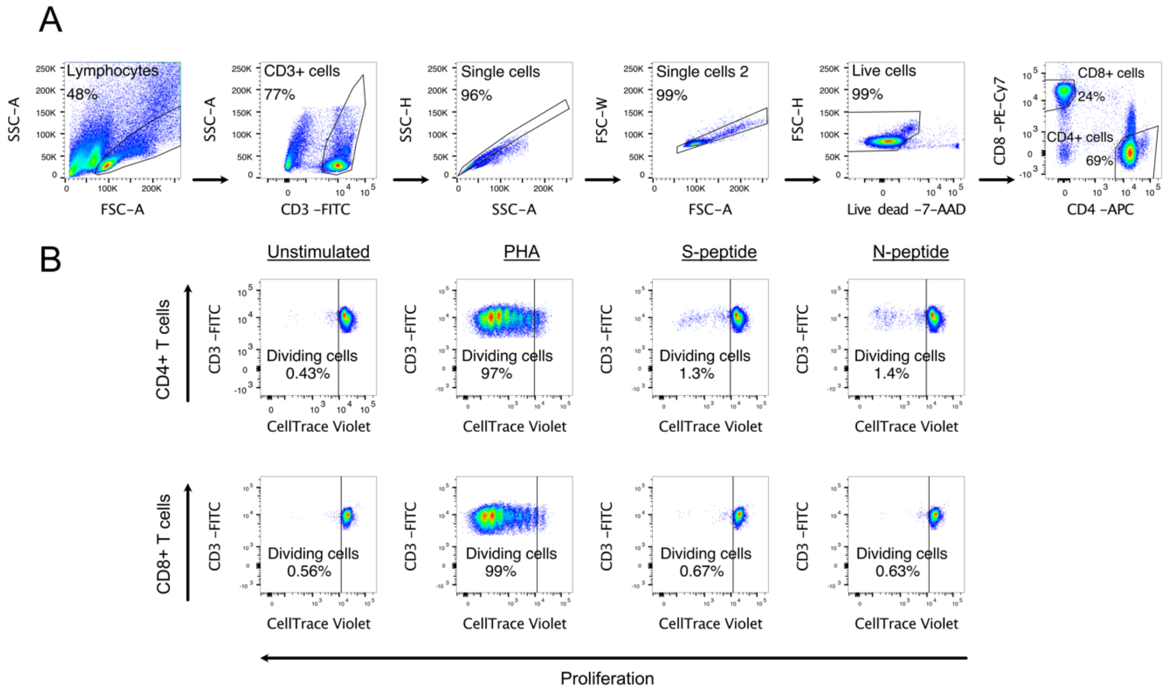

Supplemental Figure 3: Gating strategy for T-cell proliferation analysis. (A) Gating procedure, starting by gating of lymphocytes among the entire mononuclear leukocyte population, followed by gating of the CD3+ lymphocytes, gating for single CD3 lymphocytes based on two parameters (side scatter, SSC and forward scatter, FSC), gating for live cells (7-AAD negative), before identification of CD8+ and CD4+ CD3+ T cells. (B) Representative results of the percent of CD4+ and CD8+ T cells that proliferated in response to stimulation with S-peptides, N-peptides, PHA and medium alone (unstimulated). Proliferation was assessed as bleaching of Cell Trace Violet-stained CD4+ and CD8+ T cells after stimulation.

Supplemental Figure 4

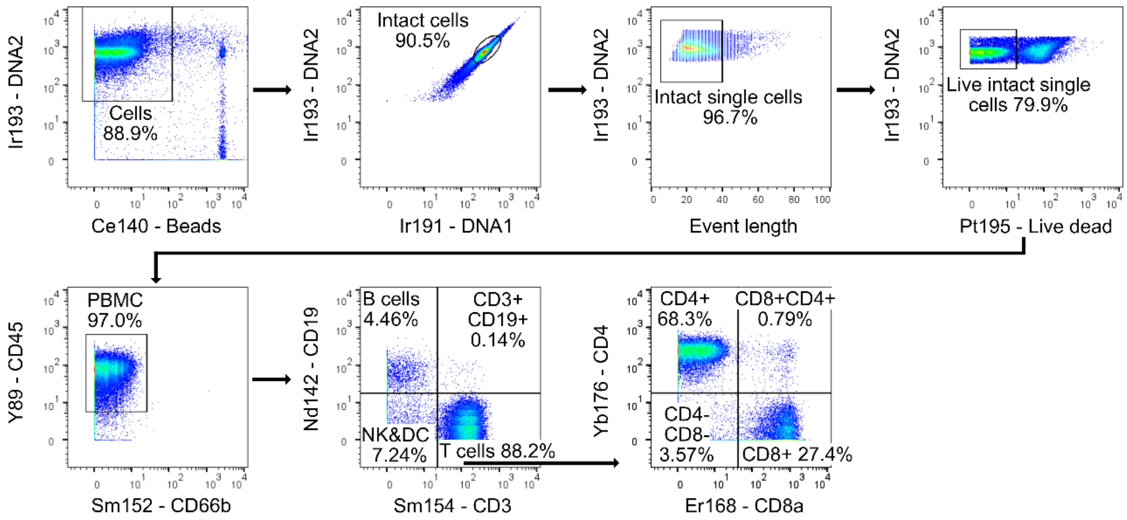

Supplemental Figure 4: CyTOF gating strategy for T cells. First, live, intact, single mononuclear leukocytes were identified based on CD45+ CD66b- expression. Next, B cells (CD19+, CD3-), NK cells and dendritic cells (CD19-, CD3-) and T cells (CD19-, CD3+) were gated based on CD19 and CD3 expression. Lastly, CD8+ and CD4+ T cell populations were gated from the CD3+ T cell gate.

Supplemental Table 1. Clinical characteristics and self-reported symptoms of study participants with IgG-dominated, IgA-dominated and negative antibody patterns.

**Antibody pattern**	**IgG** (n=38)		**IgA** (n=15)		**Negative** (n=97)	
**Clinical characteristics**						
Age	44	± 12	48	± 12	44	± 12
Female sex (%)	28	(74%)	11	(73%)	81	(84%)
BMI	25	± 5	25	± 4	24	± 4
Smoking	0		0		2	(2%)
Hypertension	3	(8%)	1	(7%)	2	(2%)
Airborne allergy	6	(16%)	3	(20%)	22	(23%)
Immunocompromised	1	(3%)	0		2	(2%)
Diabetes mellitus	1	(3%)	0		2	(2%)
Autoimmune disease	2	(5%)	3	(20%)	2	(2%)
Migraine	2	(5%)	0		7	(7%)
**Self-reported symptoms**						
Fever	17	(45%)	0		3	(3%)
Cough	28	(74%)	2	(13%)	13	(13%)
Headache	23	(60%)	5	(33%)	22	(23%)
Anosmia	18	(47%)	0		2	(2%)

Six patients out of the 156 study participants were excluded from this analysis because they provided an insufficient number of serum samples for antibody testing making it impossible to categorize an antibody pattern. Data are presented as mean ± SD for normally distributed continuous data and as total number and % for categorical data.

Supplemental Table 2. Study participants whose T cells were analyzed by mass cytometry

Study participant	Antibody pattern	T cell proliferation (CD4+ and CD8+)	IFN-γ response
B17	A + G	No	Nucleocapsid
B23	A + G	No	Nucleocapsid
F02	A + G	0.43% CD4 to nucleocapsid	Nucleocapsid
S12*	G only	No	Nucleocapsid
F12	A only	0.17-0.25% CD4 to nucleocapsid & spike	No
S17	A only	No	No
S18	Negative	0.12-0.18% CD8 to spike & nucleocapsid	No
S19	Negative	0.1% CD8 to nucleocapsid	No

*IgA-deficient individual

Supplemental Table 3

Metal-conjugated antibodies used for Helios CyTOF mass cytometry

Antibody specificity	Antibody clone	Metal conjugate
CD45	HI30	89Y
CD196[a]	G034E3	141Pr
CD19	HIB19	142Nd
CD45RA[a]	HI100	143Nd
CD38[a]	HIT2	144Nd
CD31/PECAM-1[a]	WM59	145Nd
IgD	IA6-2	146Nd
CD11c[a]	Bu15	147Sm
IgA	Polyclonal	148Nd
CD25 (IL2R)[a]	2A3	149Sm
CD138[a]	DL-101	150Nd
CD14[a]	M5E2	151Eu
CD66b	80H3	152Sm
CD62L[a]	DREG-56	153Eu
CD3	UCHT1	154Sm
CD27[a]	L128	155Gd
CD183[a]	G025H7	156Gd
CD194 (CCR4)[a]	L291H4	158Gd
FOXP3[a]	259D/C7	159Tb
CD28[a]	CD28.2	160Gd
CD69[a]	FN50	162Dy
CD56 (NCAM)[a]	NCAM16.2	163Dy
CD127 (IL-7Ra)[a]	A019D5	165Ho
CD314 (NKG2D)[a]	ON72	166Er
CD197[a]	G043H7	167Er
CD8[a]	SK1	168Er
CD24[a]	ML5	169Tm
CD152/CTLA-4[a]	14D3	170Er
Granzyme B[a]	GB11	171Yb
IgM	MHM-88	172Yb
HLA-DR[a]	L243	173Yb
CD279 (PD-1)[a]	EH12.2H7	174Yb
CD274 (PD-L1)[a]	29E.2A3	175Lu
CD4[a]	RPA-T4	176Yb
CD16[a]	3G8	209Bi

[a]Used for clustering analysis.
